# Algal oil alleviates antibiotic-induced intestinal inflammation by regulating gut microbiota and repairing intestinal barrier

**DOI:** 10.3389/fnut.2022.1081717

**Published:** 2023-01-16

**Authors:** Huimin Zhang, Zhenxia Xu, Wenchao Chen, Fenghong Huang, Shouwen Chen, Xu Wang, Chen Yang

**Affiliations:** ^1^Oil Crops Research Institute of the Chinese Academy of Agricultural Sciences, Oil Crops and Lipids Process Technology National and Local Joint Engineering Laboratory, Hubei Key Laboratory of Lipid Chemistry and Nutrition, Wuhan, China; ^2^State Key Laboratory of Biocatalysis and Enzyme Engineering, Environmental Microbial Technology Center of Hubei Province, Hubei University, Wuhan, China; ^3^Institute of Food & Nutrition Science and Technology, Shandong Academy of Agricultural Science, Jinan, China; ^4^College of Animal Science and Technology, Huazhong Agricultural University, Wuhan, China

**Keywords:** algal oil, polyunsaturated fatty acids, intestinal barrier, intestinal inflammation, gut microbiota

## Abstract

**Introduction:**

Taking antibiotics would interfere with gut microbiota and increase the risk of opportunistic pathogen infection and inflammation.

**Methods:**

In this study, 36 male C57BL/6 mice were divided into 4 groups (*n* = 9) to investigate whether two kinds of algal oil could alleviate the intestinal damage induced by CS (Ceftriaxone sodium). These algal oils were obtained from *Schizochytrium* sp. cultures using Yeast extract (YE) and Rapeseed meal (RSM) as substrate, respectively. All tested mice were administrated with CS for 8 days and then the colon pathological morphology, the expression levels of inflammatory factors and the gut microbial profile were analyzed in mice supplemented with or without algal oil.

**Results:**

The results showed that both YE and RSM algal oils markedly reduced mucosal damage and intestinal inflammatory response in CS-treated mice by inhibiting the pro-inflammatory cytokine tumor necrosis factor (TNF)-α, interleukin (IL)-6 and myeloperoxidase (MPO) activity. In addition, fluorescence immunohistochemistry showed that the tight junction protein ZO-1 was increased in mice supplemented with YE and RSM algal oil. Furthermore, YE algal oil promoted the beneficial intestinal bacteria such as *Lachnospiraceae* and S24_7 compared with the CS group, while supplementation with RSM algal oil enriched the Robinsoniella. Spearman’s correlation analysis exhibited that *Melissococcus* and *Parabacteroides* were positively correlated with IL-6 but negatively correlated with IL-10.

**Discussion:**

This study suggested that supplementation with algal oil could alleviate intestinal inflammation by regulating gut microbiota and had a protective effect on maintaining intestinal barrier against antibiotic-induced damage in mice.

## 1. Introduction

Mammalian gastrointestinal tract contains an extremely diverse and complex microbial community, which plays an important role in maintaining host health (1, 2). Taking antibiotics would interfere with the gut microbiota and increase the risk of opportunistic pathogen infection, diarrhea and inflammation (3–5). Short-term antibiotic therapy can convert the intestinal flora to a long-term dysbiotic state, which may promote the development and progression of the disease (6). Ceftriaxone sodium, a common antibiotic, has been shown would decrease the microbial diversity, induce the activation of T cells and B cells in the intestinal associated lymphoid tissue and reduce the mesenteric lymph nodes and the intestinal secretion of slgA (secreted immunoglobulin A) after oral administration (7). Long-term use of ceftriaxone sodium will not only lead to intestinal flora disorders, but also cause intestinal tissue lesions, growth inhibition and affect immune defense and immune regulation (8, 9).

The intestinal microbiota maintains a symbiotic relationship with the host and regulates a number of important functions, including immunity, host metabolism and intestinal barrier function (10). Individual differences of intestinal flora plays an important role in maintaining internal homeostasis and internal environmental stability through its metabolites (11). The intestinal microbiota produce a variety metabolites, some of which could serve as messengers to alter the gut microbiota, thereby affecting various disease states (12). Several reports have suggested that the gut microbiota is an important driver for inflammation in inflammatory bowel disease (IBD), with the perturbation of gut microbiota disrupting the gut barrier, promoting intestinal permeability, and activating the immune system (13). Gut dysbiosis, especially the loss of key intestinal flora that has the function of regulating intestinal inflammation, has been widely demonstrated as the causes of impaired homeostasis within the intestinal mucosa (14, 15).

Recently, it has been found that the gut microbiota is closely related to human health and is influenced by the environment, diet and antibiotics (16, 17). Among them, diet is considered to be an effective pathway to restore immune homeostasis and intestinal dysbiosis to mitigate the side effects of antibiotics (18, 19). Nutrients had a profound effect on gut microbes and gut immunity, which were mediated by gut microbes, and there is a strong correlation between these factors (20). Previous studies have shown that antibiotic-induced disturbances of the intestinal barrier and intestinal microbiota can be modulated by supplementation with n-3 PUFA polyunsaturated fatty acids (PUFAs) such as algal oil and fish oil (21, 22). Krill oil has been reported to increase species richness and modulate microbial interactions to restore microbial dysbiosis in models of infection-induced colitis (23). Several studies have also suggested that fish oil supplementation is a potential strategy for the treatment of IBD, and it can prevent or reduce gastrointestinal disorders caused by experimental colitis (24, 25). Additionally, other studies demonstrated that camellia oil intake increased the alpha diversity, the ratio of Firmicutes/Bacteroidetes and abundance of *Bifidobacterium*, while also decreased the abundance of *Prevotella*, which could well improve acetic acid-induced colitis (26).

n-3 PUFAs, in particular docosahexaenoic acid (DHA) and eicosapentaenoic acid (EPA), which have diverse health benefits including to improve metabolic homeostasis and immune cell function as dietary supplements for human beings and animals (27, 28). Fish oil is a common, well-known dietary n-3 polyunsaturated fatty acid (PUFA) dietary supplements originated from marine fish (29). And the total content of n-3 PUFA in fish oil is usually less than 30% (30), and it is liable to be highly oxidized, thus do not meet the label content of n-3 PUFA (31). Plant based algal oils typically contains 50–55% higher n-3 PUFA than that of fish oil (32). Oils rich in EPA and DHA can be produced directly from microalgae by fermentation technology, and algal oil produced from *Schizochytrium* sp. have been developed as a sustainable source of DHA (33). Currently, studies have shown that algal oil rich in DHA not only has antitumor and anti-inflammatory activities (34), but also can markedly improve the intestinal barrier by reducing proinflammatory factors and regulating intestinal flora and metabolites in DSS induced colitis (35).

Algal oil product was extracted from *Schizochytrium* sp. using yeast extract (YE) as fermentation substrate in industry. Our previous study have shown that rapeseed meal (RSM) was a potential nitrogen source to positively improve the DHA accumulation in marine dinoflagellate *C. cohnii* culture (36). However, whether these two kinds of algal oil have benefits effects on the antibiotic treated mice remains unknown. In this study, we evaluated the effects of algal oil fermented by YE and RSM on the intestinal injury induced by CS, and also investigated the association with the modulation of gut microbiota and reparation in intestinal barrier.

## 2. Materials and methods

### 2.1. Algal oil preparations

*Schizochytrium limacinum* SR21 (*S. limacinum* SR21) strain was obtained from American Type Culture Collection (ATCC MYA-1381), which was cultivated with yeast extract and rapeseed meal as nitrogen sources. 5% (v/v) cells were added into fermentation medium [glucose (50 g/L), sodium glutamate (30 g/L), artificial sea salt (20 g/L) and yeast extract or rapeseed meal (10 g/L)], and inoculated at 28°C for 48 h. After the fermentation, the bacteria were collected, freeze-dried. The algal oil was extracted from chloroform methanol according to the method of Fattah et al with a modification **(37)**. The fatty acid composition of these two algal oils was determined by GC and shown in Supplementary Table 1.

### 2.2. Chemicals

The MPO (myeloperoxidase) kit was purchased from Nanjing Jiancheng Biological Engineering Institute (Nanjing, China). The BCA (bicinchoninic acid) protein concentration kit and MDA (malonaldehyde) kit were purchased from Beyotime Biotechnology (Shanghai, China). TNF-α, IL-10, IL-6, and IL-1β enzyme-linked immunosorbent assay (ELISA) kits were purchased from Elabscience Biotechnology Co., Ltd. (Wuhan, China). All other chemicals used were reagent grade.

### 2.3. Animal experiments

**FIGURE 1:**
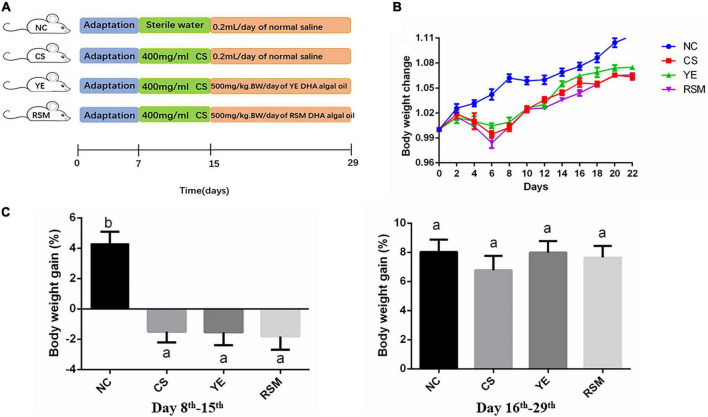
Effect of two kinds of algal oil on clinical symptoms in CS-treated mice. **(A)** Scheme of the animal experimental design. **(B)** Body weight change during the experiment. **(C)** Body weight gain on day 8th–15th and day 16th–29th. Data (*n* = 9 per group) are expressed as mean ± SEM; groups with different letters statistically differ. Groups with the same letter are considered to have no significant differences, groups with different letters are significantly different from each other (*P* < 0.05).

A total of 36 male C57BL/6 mice with average body weight 19 g were provided by Experimental Animal Center of HuaZhong Agricultural University. This animal experiment was approved by the Animal Ethics Committee of Huazhong Agricultural University (Permission HZAUMO-2021-0170). The mice were subjected to 1-week acclimation in a SPF animal chamber with humidity of 50 ± 5%, temperature of 23 ± 2°C, dark/light cycle for 12 h, under food and water *ad libitum*. Two kinds of algal oil were dissolved in 0.1% flaxseed gum solution and administered to mice by gavage (38). The experimental design was referred to Xu et al. with small modification (Figure 1A) (35). The mice were randomly divided into 4 groups (*n* = 9) within three mice per cage: negative control group (NC), CS group, YE group, and RSM group. Mice were fed adaptively for one week, then NC group was given normal saline 0.2 ml/d once a day for 22 days. CS, YE, and RSM groups were administered 0.2 ml of CS (400 mg/ml) intragastrically once a day for 8 days to induce intestinal dysbacteriosis. YE group and RSM group were given 500 mg/kg⋅bw/d of YE algal oil or RSM algal oil for consecutive 14 days after CS administration, while CS group was given normal saline 0.2 ml/d during this time.

The diet was listed in Supplementary Table 2. The dosages of algal oil and CS were referred to yang et al. (39). Mice were executed and colonic tissue was scissored from the mice, and fresh feces were collected by gently squeezing the colon in clean bench. Fecal particles from each mouse were then immediately stored in a refrigerator at −80°C until DNA was extracted for bioinformatics analysis. The colon tissues were separated and stored at −20°C for further analysis.

### 2.4. Assessment of colitis symptoms

Body weight, stool consistency and blood in stool were observed and recorded every day. Hematoxylin and eosin (H&E) staining and fluorescent immunohistochemistry were done by Wuhan Pinofair Company (Wuhan, China). The distal colon tissue of 0.5 cm mice was fixed with 4% paraformaldehyde solution, then hematoxylin and eosin (H&E) staining was performed, and the colon tissue was observed under a light microscope for histological examination. Colon mucosal damage was scored, using the following parameters: inflammatory cell infiltration (rare inflammatory cells in the lamina propria, 0; increase in the number of inflammatory cells in the lamina propria, 1; inflammatory cells extending into the submucosa, 2; infiltration across the wall, 3) and tissue damage (no mucosal damage, 0; discrete lymphoepithelial damage, 1; surface mucosal erosion, 2; extensive mucosal damage and extension through deep structures of the intestinal wall) (40). MDA content and MPO activity in colon tissue were measured by Beyotime Biotechnology (Shanghai) and MPO assay (Nanjing Jiancheng Institute of Biological Engineering), respectively. The levels of inflammatory cytokines in colon (IL-10, IL-1β, IL-6, and TNF-α) were detected by ELISA kit (Elabscience Biotechnology Co., Ltd., Wuhan, China). The test methods were carried out according to the kit instructions.

### 2.5. Immunohistochemistry analysis

Immunofluorescence assay was performed according to the reported method and the colon tissues were immobilized in pure acetone and permeated at –20°C (41). Then the tissues were cultured with primary antibodies overnight at 4°C, followed by incubation with fluorescein isothiocyanate (FITC) labeled secondary antibodies for 1 h at room temperature. Finally, the sections were counterstained with 4,6-diamidino-2-phenylindole (DAPI). FITC and DAPI images were taken from the same area of tissue section, and Image pro plus 6.0 software was used for quantitative analysis of fluorescence.

### 2.6. Bioinformatics analysis

The analysis of bacterial community structure was performed by 16S rRNA gene sequence in the V3-V4 region via the Illumina HiSeq platform by Personalbio in Shanghai. The amplified products were sequenced of Illumina MiSeq platform, and 97% of the similar sequences were divided into operational taxa (OTU). QIIME software was used to reflect the species diversity and richness of intestinal microflora through α diversity index. To calculate alpha diversity (in sample), we sparse the OTU table and calculate the richness index based on the genus spectra of four different groups. The Alpha diversity index was Chao1, Simpson, Shannon, and Pielou_e indices. For beta diversity (between samples), a weighted UniFrac distance matrix is generated using the OTU table, and principal coordinate analysis (PCoA) is performed with the ggplot2 package in R and displayed. We used linear discriminant analysis effect size (LEfSe) to analyze the significant differences in relative abundance of groups among the four groups. Groups with linear discriminant analysis (LDA) values greater than 2 and *P* < 0.05 were considered to be significantly enriched. The software PICRUSt predicts the function of the gut microbiota based on 16S rRNA gene data. The 16S rRNA signature sequences were compared with the reference sequences to construct a new evolutionary tree. Using the Castor cryptic prediction algorithm, the nearest sequence species of the feature sequence was inferred from the copy number of the gene family corresponding to the reference sequence in the evolutionary tree, and then the presence of metabolic pathways was inferred using MinPath to obtain data on the abundance of metabolic pathways in each sample.

### 2.7. Statistical analysis

All data were reported as mean ± standard error of the mean (SEM). One-way analysis of variance (ANOVA) followed by Tukey *post-hoc* analysis was used in assessing the differences between the two groups. For non-parametric variables, data were examined by non-parametric Kruskal–Wallis test followed by Student–Newman–Keuls (SNK) test. All other statistical tests were performed using the GraphPad Prime 8 (GraphPad Software, San Diego, CA, USA). *P* < 0.05 was considered statistically significant.

## 3. Results

### 3.1. Two kinds of algal oil ameliorated CS-induced intestinal injury

During the whole experiment, body weight, diarrhea and hematochezia of the mice were observed and recorded daily. Figure 1B has shown the changes of body weight in mice on day 8th. The body weight of NC mice increased gradually during the experiment, while the body weight of CS mice continued to decrease during the first 6 days after the CS consumption, and began to increase from the 8th day. As depicted in Figure 1C, during the 7 days after CS treatment, the body weight of CS, YE and RSM mice all decreased (*P* < 0.05), and there was no significant difference in weight gain during the subsequent 14 days recovery period (*P* > 0.05).

### 3.2. Algal oil alleviated CS-induced colonic tissue injury

**FIGURE 2:**
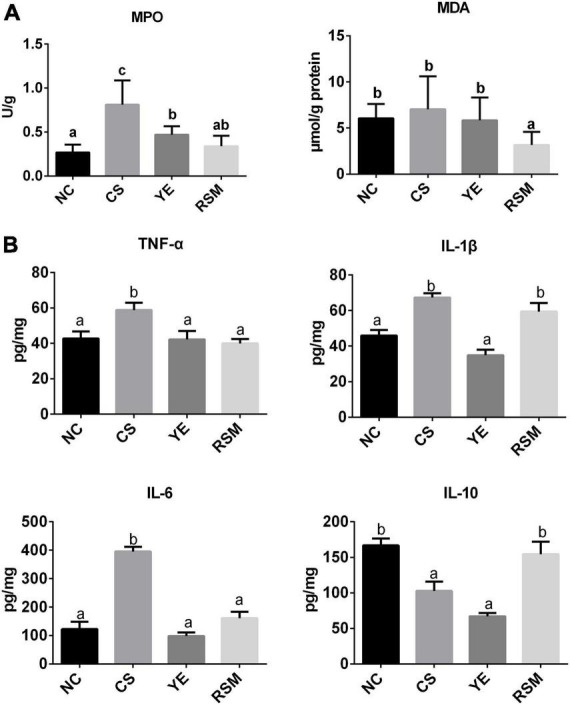
Effects of two algal oils on MDA, MPO **(A)** and cytokines levels **(B)** in the colon tissue for the tested mice. Data (*n* = 9 per group) are expressed as mean ± SEM; groups with different letters statistically differ. Groups with the same letter are considered to have no significant differences, groups with different letters are significantly different from each other (*P* < 0.05).

To evaluate the effects of two algal oils on colonic oxidative stress, MDA content and MPO activity were determined. As it was shown from in Figure 2A, MPO concentration in CS mice was markedly higher than that in NC mice (*P* < 0.05), and it was reduced in both YE mice (*P* < 0.05) and RSM mice (*P* < 0.05) compared to CS group. The MDA activity in CS mice did not increase significantly compared to the NC mice. In addition, to define whether the two algal oils reduced colonic inflammation in mice, the levels of pro-inflammatory cytokines IL-1β, TNF-α, IL-6, and anti-inflammatory cytokines IL-10 were also measured (Figure 2B). As expected, CS treatment caused a significant increase in the level of pro-inflammatory cytokines TNF-α, IL-1β, and IL-6 compared with mice in NC group (*P* < 0.05). Both algal oils YE (*P* < 0.05) and RSM (*P* < 0.05) reduced TNF-α level after CS administration, as well as IL-6. Conversely, it was the supplementation of YE but not RSM algal oil reduced IL-1β level. RSM algal oil also increased the anti-inflammatory factor IL-10 level compared with CS mice (*P* < 0.05).

### 3.3. Algal oil protected the intestinal barrier

**FIGURE 3:**
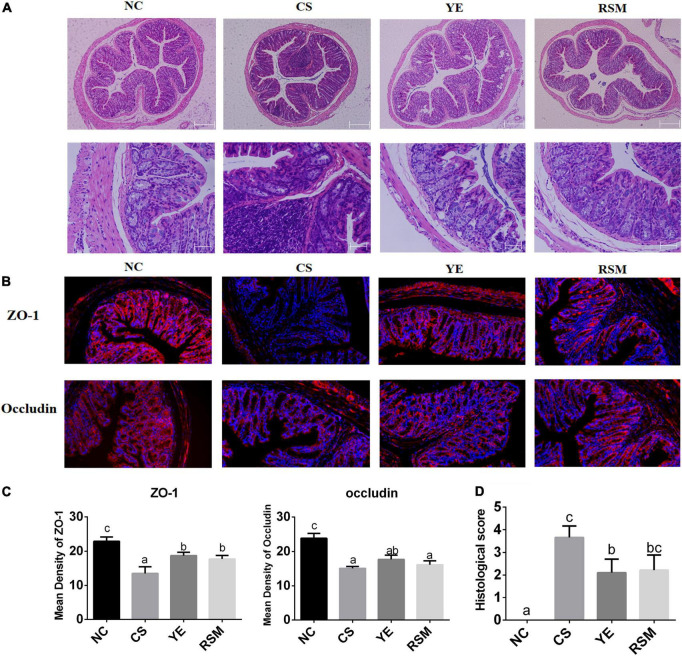
Effects of two algal oils on intestinal barrier damage caused by CS. **(A)** H&E staining of representative colons from untreated mice or mice treated with CS and algal oil are shown using the same microscope (magnification of 100×, 400×). **(B)** Representative immunofluorescence staining of the tight junction proteins in the colon tissue (200×). **(C)** The mean density of the tight junction proteins (ZO-1, Occludin). **(D)** Histological score. Data (*n* = 9 per group) are expressed as mean ± SEM; groups with different letters statistically differ. Groups with the same letter are considered to have no significant differences, groups with different letters are significantly different from each other (*P* < 0.05).

It was found in Figure 3A that the colic mucosa of CS mice was extensively eroded and edema, and the superficial epithelial cells and goblet cells were disappeared. Submucosa were extensively infiltrated by inflammatory cells, and crypt rupture and loss. In NC group, the colonic tissue was normal, the colonic mucosa was intact, the intestinal wall was neat, and the crypt structure was healthy. Compared with CS mice, CS-induced inflammatory infiltration and crypt damage have been reduced in YE mice and RSM mice and the mucosal structure has been partially restored.

Figure 3B displayed the fluorescent immunohistochemical results of colon tissue. To evaluate the effect of two algal oils on intestinal barrier in mice, we detected the protein levels of Occludin and Zonula occludens-1 (ZO-1) in colon tissues. In terms of imaging results, DAPI (blue) stained the nucleus and tight junction proteins appeared red. Immunofluorescence assays demonstrated considerable loss of ZO-1 and Occludin in tight junction complexes and structural discontinuities in the inner lining of the colonic epithelium in CS mice. It can be seen that the tight junction proteins of NC mice was quite clear. Tight junction proteins were increased in both YE mice and RSM mice after supplementation of algal oil. Figure 3C showed that the fluorescence density of tight junction proteins ZO-1 and Occludin. Compared with the NC mice, two tight junction proteins were significantly reduced in the CS mice (*P* < 0.05). The tight junction protein ZO-1 was increased in both YE and RSM mice compared to CS mice (*P* < 0.05), while Occludin protein was not significantly increased (*P* > 0.05). As depicted in Figure 3D, histological tissue scores were higher in the CS mice compared to the NC mice and significantly decreased after supplementation of YE algal oil (*P* < 0.05).

### 3.4. Two kinds of algal oil modulated the intestinal microflora

**FIGURE 4:**
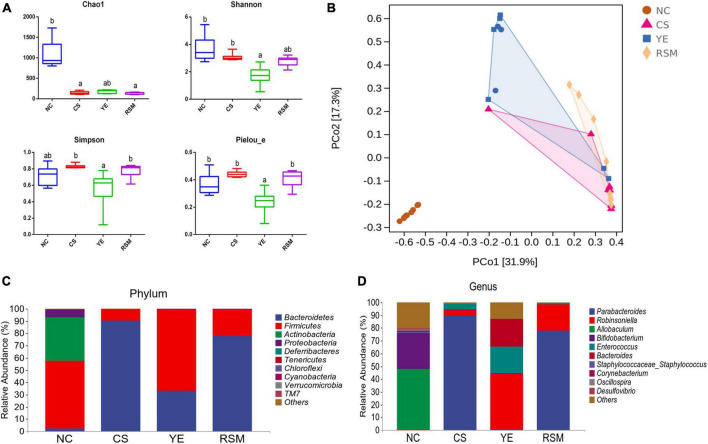
Effects of two kinds of algal oil on the intestinal microbiota of CS induced mice. **(A)** Alpha diversity indexes of the gut microbiota. **(B)** PCoA diagram based on the bray-Curtis distances of beta diversity of the gut microbiota. **(C)** Gut microbial compositions at phylum level (only top 10 abundant phylum were included). **(D)** Gut microbial compositions at genus level (only top 10 abundant genus were presented). Data (*n* = 9 per group) are expressed as mean ± SEM; groups with different letters statistically differ. Groups with the same letter are considered to have no significant differences, groups with different letters are significantly different from each other (*P* < 0.05).

As shown in Figure 4A, the NC mice had the highest Chao1 index compared with the other groups, and there was no significant difference among the mice supplemented with two algal oils or the CS group mice (*P* < 0.05). The Simpson index and Pielou_e index in RSM mice was higher compared to YE mice (*P* < 0.05). Shannon index in YE mice was decreased compared with CS mice, and there was no significant difference between YE mice and RSM mice (*P* < 0.05). Beta diversity was assessed using the Bray-Curtis distance for PCoA. PCoA diagram showed that the microbial structure of CS mice was significantly different from NC mice. In addition, mice supplemented with YE algal oil and RSM algal oil also displayed significant differences in microbial composition compared with CS mice (Figure 4B). Intergroup differences based on weighted Unifrac distance matrix analysis also suggested groups separated from each other (Supplementary Figure 1C).

At the phylum level, the top 10 phylum were selected and shown in Figure 4C. Bacteroidetes, Firmicutes, and Actinobacteria were top three dominated phylum in the gut microbiota. Firmicutes and Actinobacteria were the richness taxa in NC mice. After CS treatment, Bacteroidetes was up-regulated (*P* < 0.05), while Firmicutes was down-regulated (*P* < 0.05) when compared with the NC mice. The abundance of Firmicutes was increased in YE mice, while it did not change in RSM mice. At the genus level, the top 10 taxa were also analyzed, and Figure 4D showed significant changes in the measured genera. In NC mice, *Allobaculum* was the most abundant genus, followed by *Bifidobacterium*, while the most abundant genus in CS mice was *Parabacteroides*. *Robinsoniella* was the most common genus in YE mice, while *Parabacteroides* was the most common genus in RSM mice (*P* < 0.05).

**FIGURE 5:**
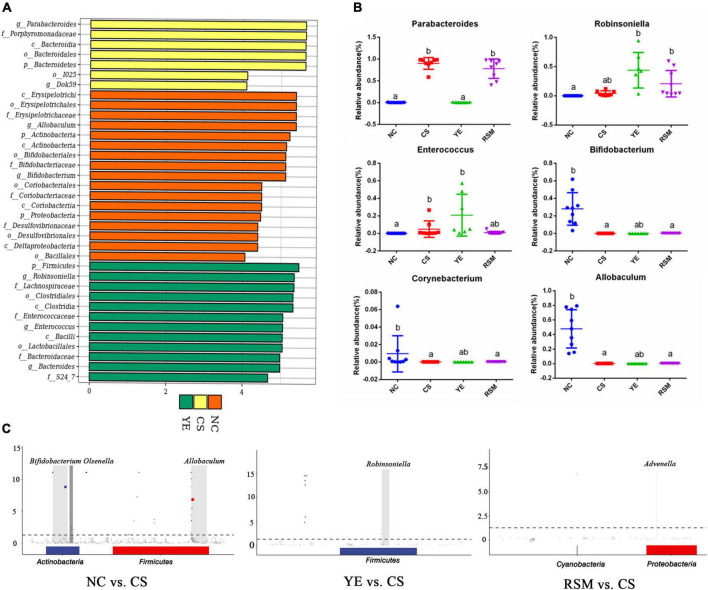
Effects of two kinds of algal oil on gut microbiota profile. **(A)** Histogram of the LDA value and clade distribution for the differential microbial community in each group. **(B)** The relative abundance of representative bacteria at genus level. **(C)** ASV/OTU Manhattan chart based on metagenomeSeq differences. Data (*n* = 9 per group) are expressed as mean ± SEM; groups with different letters statistically differ. Groups with the same letter are considered to have no significant differences, groups with different letters are significantly different from each other (*P* < 0.05).

Linear discriminant analysis (LDA) was used to identify statistically significant biomarkers and reveal the dominant microorganisms in each group. As shown in Figure 5A, there were 7, 17, and 12 taxonomic groups at different taxonomic levels enriched in NC group, CS group and YE group, respectively. CS group has enriched the taxa of *Parabacteroides* and *Porphyromonadaceae*, YE group has enriched the taxa of *Firmicutes*, RSM group has no specific biomarkers. Figure 5B has shown more information about the top six groups with significant changes in gut microbiota. Compared with NC group, *Parabacteroides* and *Enterococcus* were increased in CS group (*P* < 0.05), while *Allobaculum*, *Bifidobacterium* and *Corynebacterium* in CS group were decreased (*P* < 0.05). *Robinsoniella* and *Enterococcus* in YE group were higher than those in NC group (*P* < 0.05). Figure 5C showed an ASV/OTU Manhatton diagram based on metagenomic differences. Each dot or circle in the coordinate system represents one ASV/OTU, and the size represents its relative abundance. The relative abundance of NC group and CS group was significantly different, and the microbiota of YE group and RSM group were increased compared with CS group, and the relative abundance of the two groups was also different.

**FIGURE 6:**
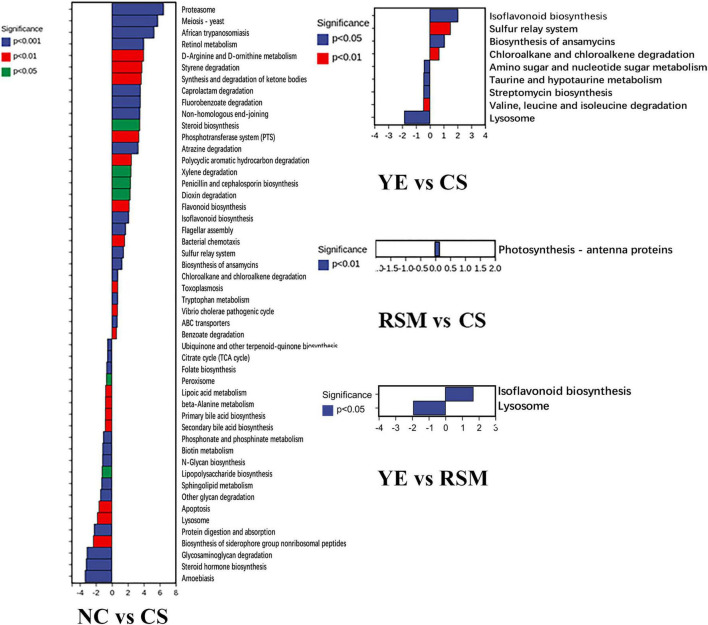
Changes in metabolic pathways of CS and two algal oils administered. Significant differences are indicated as *P* < 0.05, *P* < 0.01, *P* < 0.001.

As depicted in Figure 6, PICRUSt was carried out to predict the function of the KEGG category represented by algal oil treated mice. These metabolic pathways include microbial gene functions related to cellular processes, genetic information processing, metabolism, environmental information processing, biological systems, and human disease (Supplementary Figure 2). These changes related to metabolic disorders and inflammatory responses. The different metabolic pathways between NC group and CS group was most significant (*P* < 0.05, *P* < 0.01, *P* < 0.001), and there were 50 different metabolic pathways between the two groups such as Proteasome and Meiosis—yeast. There were nine different metabolic pathways between YE group and CS group (*P* < 0.05, *P* < 0.01), Photosynthesis—antenna proteins was the only metabolic pathway that was different between RSM group and CS group (*P* < 0.05). Two different metabolic pathways exist between the YE group and RSM group (*P* < 0.05).

### 3.5. Changes in the composition of intestinal flora were associated with the colon inflammation index

**FIGURE 7:**
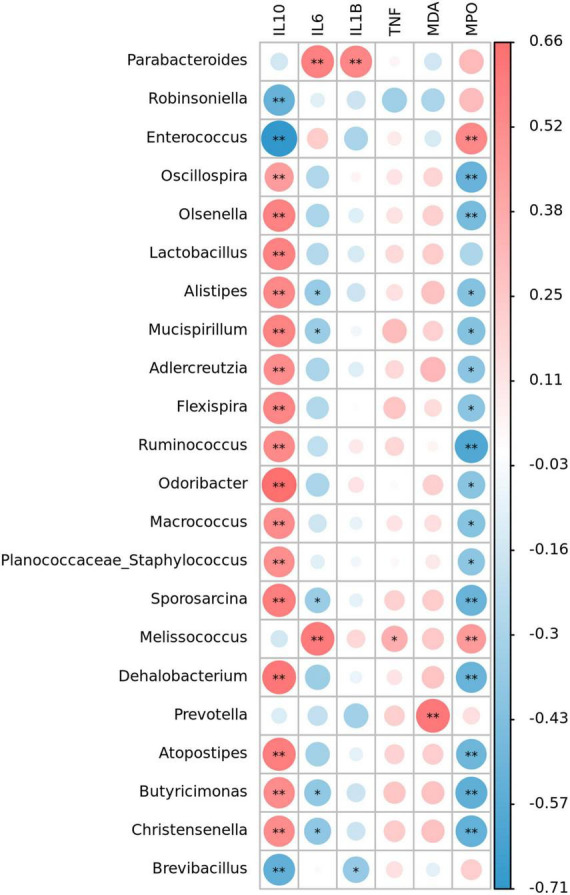
Heat map representation of the Spearman’s correlation coefficient between bacterial taxa and colitis-related index (IL-10, IL-6, IL-1β, TNF-α, MDA, and MPO). Positive correlation is represented in red and negative correlation is represented in blue. Significant differences are indicated as *P < 0.05, ^**^P < 0.01.

Spearman correlation analysis was used to evaluate the correlation between significantly changed groups in intestinal microbiome and colitis correlation index. Figure 7 showed that 22 OTUs were negatively or positively correlated with at least one biochemical index (*P* < 0.05, marked with *). Oxidative stress index was associated with inflammatory responses, but there was little correlation among gut microbiota and MDA and TNF-α level. More than a dozen of microbial were positively correlated with IL-10 and negatively correlated with IL-6. Sixteen species including *Lactobacillus*, *Alistipes*, and *Mucispirillum* were negatively correlated with IL-10, and eight species including *Oscillospira*, *Sporosacina*, and *Ruminococcus* were significantly negatively correlated with MPO (*P* < 0.05).

### 3.6. Correlation between fatty acid composition of algal oil and gut microbiota

**FIGURE 8:**
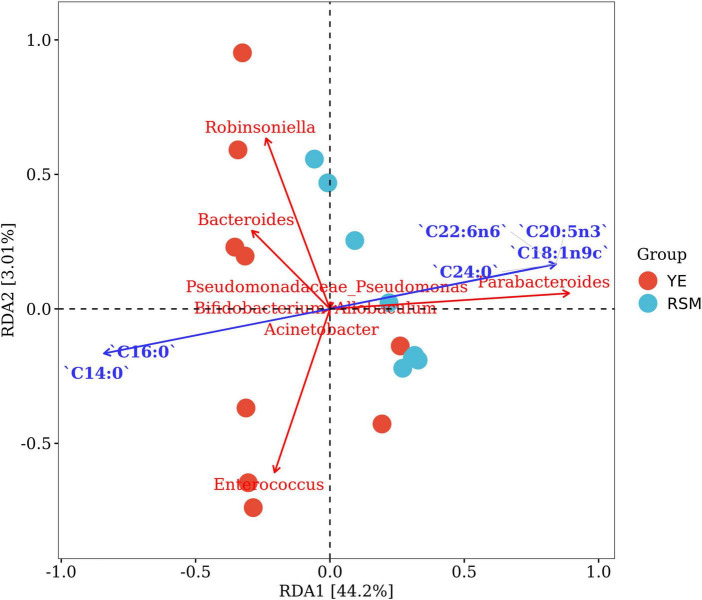
Redundancy analysis (RDA) showed the correlation between changed gut microbiota and fatty acid composition of two algal oil. The red dots and blue dots represent the YE group and RSM group, respectively. The blue arrow indicates a significant correlation with the bacteria (*P* < 0.05).

Redundancy analysis (RDA) was performed to explore the correlations between changed gut microbiota and fatty acid composition from two algal oils. As shown in Figure 8, 6 majority fatty acids and the top 10 bacterial genera in gut microbiota were used for correlation analysis. The results showed that RDA1 and RDA2 explained 44.2 and 3.01% of the total variance for fatty acid composition and gut microbiota. The results indicated a close correlation of top six fatty acids with on gut microbiota, and two saturated fatty acid (C14:0 and C16:0) showed negative correlation with the other four fatty acids. Unsaturated fatty acid including n-3 PUFA (C22:6n6 and C20:5n3) contributed significant impacts on *Parabacteroides* and *Allobaculum*. While saturated fatty acid C14:0 and C16:0 fatty acids demonstrated correlation with the *Bacteroides* and *Enterococcus* genus.

## 4. Discussion

Intestinal flora is closely related to the health of host, and the dysbiosis of gut microbiota would cause certain adverse effect on host health (42). Previous studies have shown that antibiotics consumption would induce long-lasting deleterious effects for host and perturb commensal microbial communities (43, 44). The DSS colitis models has been widely used to evaluate the effect of many natural biocompound on UC (ulcerative colitis) in humans (45). However, there were few studies on antibiotics induced intestinal barrier damage in mice. It has been investigated that ceftriaxone pretreatment can significantly reinforce inflammation and disrupt the balance of the gut microbiota (46). In this study, we used ceftriaxone sodium to induce intestinal injury in mice and explored the reparation effect of two algal oils fermented with different substrates in mice.

Different nitrogen sources affect the fatty acid composition of algal oil product, which resulted in different gut microbiota profile in algal oil supplemented mice. n-3 PUFAs exert positive health effects in several biological processes, including immune regulation, making them potential therapeutic agents against inflammatory diseases (47). According to our results, although CS did not induce serious colitis, it caused extensive erosion and edema of the colonic mucosa, and loss of crypt rupture. This result was consistent with the previous study that reported disruption of the intestinal microbial barrier due to ceftriaxone sodium (48). Antibiotics induced immune dysfunction resulted in disproportionate release of cytokines and increased intestinal mucosal inflammation (49), including higher expression levels of IL-1β, IL-6, and TNF-α, which were considered as common biomarkers in various inflammatory conditions (50). Here, both two algal oils contain high content of DHA and EPA, they showed benefit effect on immune response by inhibiting the expression and secretion of TNF-α and IL-6 in colon tissue. The activity of MPO was also decreased after administration of two algal oils, which might be related to the presence of PUFA fatty acid inhibited the secretion and expression of inflammatory factor cells. However, there is a limitation that we did not test the serum inflammatory factors (IL-1β, IL-6, and TNF-α) in CS treated mice, which will be investigate in our future study.

n-3 PUFA oil consumption was associated with improved intestinal barrier function (51, 52). Tight junction (TJ) complexes restricted and regulated intestinal permeability, of which, the ZO-1, Occludin, and Claudin-1 were the key proteins (53, 54). Here, supplementation of two kinds of algal oil upregulated the concentration of tight junction protein ZO-1 by fluorescence quantitative analysis, while the concentration of Occludin increased insignificantly. It has been reported that TJ protein integrity strengthen the intestinal barrier, thereby limit the entry of antigens into antibodies and prevent adverse immune reactions (55). Previous experimental and clinical studies have shown that down-regulation of ZO-1, Occludin, and Claudin-1 were related with the increased intestinal permeability in IBD patients (56). Occludin, a key member of transmembrane protein, mainly regulate the permeability of TJs and maintain the polarity of epithelial cells (57). Consistent with previous studies (58), the protein concentration of Occludin and ZO-1 decreased after CS treatment in this experiment. Occludin protein did not display significant differences between CS group with YE or with RSM group, while both two kinds of algal oil improved the expression of ZO-1 in colon tissue compared with CS group. This may be due to the ability of ZO-1 protein regulates epithelial cell proliferation, mitotic spindle orientation, and mucosal repair. On this account, supplementary of algal oil might be an effective treatment to improve the concentration of some tight junction protein.

Intestinal flora is closely related to the intestinal barrier, since intestinal dysbiosis alters the intestinal mucus layer, disrupts TJs, increases intestinal permeability, and promotes bacterial translocation (59). Therapeutic strategies aimed at restoring normal flora structure and balancing microbial homeostasis can significantly enhance the intestinal barrier. It has been reported that probiotics activate the MAPK signaling pathway, prevent the loss of TJs and E-Cadherin proteins, and reduce intestinal and external permeability (60). In our study, both two kinds of algal oil partially repaired intestinal damage, suggesting that algal oil may protect the intestinal barrier by regulating intestinal microbes and promoting microbial metabolism. Administration of YE algal oil promoted the proliferation of several beneficial bacteria such as *Lachnospiraceae* and *Lactobacillales*, which were negatively correlated with epithelial cell apoptosis and intestinal IL-6 levels (61). These beneficial bacteria may further play an active role in maintaining homeostasis in the intestine by supporting epithelial cell proliferation and promoting epithelial barrier function via producing short branched-chain fatty acids.

Ceftriaxone sodium was reported to induce intestinal dysbacteriosis including beneficial bacteria and overall and local immune function decreased (7). Besides, long term ceftriaxone treatment reduced the microbial diversity but increased single bacterial species in the mouse intestine, which on 30th day and 60th day, *Robinsoniella* and *Enterococcus* genus became dominant, respectively (8). In this experiment, the consumption of CS also caused changes in microbial community structure, significantly reduced microbial diversity of index Chao1. The microbial community richness and diversity were reduced in CS mice compared with the NC mice, and the abundance of representative microorganisms was also altered by algal oil treatment. However, it is a limitation that we did not compare the gut microbiota composition after CS administration on day 15th.

Firmicutes and Bacteroidetes were the main microbiota in the intestinal tract of mice, and their abundance changes were related to the pathogenesis of obesity (62), diabetes (63), gastrointestinal cancer and stress (64). In many works, reduced F/B has been linked with improved metabolic health indicators, but recent studies showed little effect of the F/B ratio on obesity and a decreased F/B ratio was also not observed in all IBD cases (65). Firmicutes and Actinobacteria were the dominant phylum in the NC group in our study. The abnormally high proportion of Actinobacteria in NC group was due to the high abundance of *Bifidobacterium*. *Bifidobacterium* is a lactic acid producing probiotic that is abundant in newborn intestine and has many benefits for infants and children, such as nutritional, immune and anti-infective effects (66, 67). Studies have reported that Firmicutes and Bacteroidetes are dominant phylum in the normal intestine (65), but in our study NC mice had a normal diet and physiological condition during the experiment and did not behave abnormally compared to NC mice in previous experiments. We speculated that the significant enrichment of Bifidobacterium in NC mice may be related to the specific intestinal status of this test group of mice at birth, as reports have shown that *Bifidobacterium* is very sensitive to antibiotics (68, 69), which is also consistent with our results, since the abundance of *Bifidobacterium* was significantly reduced in the other three groups treated with antibiotics. After CS treatment, Bacteroidetes were up-regulated, while Firmicutes were down-regulated. YE significantly increased the composition of Firmicutes. In these cases, although algal oil intake was not able to modulate the F/B ratio, it did induce changes in some key bacteria that may be related to gut barrier protection. Compared with other groups, CS group was more enriched in *Parabacteroides* and *Porphyromonadaceae*, which can be considered as a potential biomarker for microbiota dysbiosis (70). Previous studies have shown that *Parabacteroides* was a bacteria involved in biochemical processes in many human diseases, and that *Parabacteroides* was clearly associated with IBD in many other diseases (70). Reversely, in recent studies, oral administration of *Parabacteroides* distasonis antigen was found to reduce experimental murine colitis by modulating immune and microbiota composition (71), which may need to be further investigated. YE group was enriched with Firmicutes, *Lachnospiraceae*, *Lactobacillales*, *S24_7*, and other microorganisms considered as beneficial bacteria, which were found to regulate host metabolism and immune response (72). According to studies, *S24-7* could promote the release of extracellular DNA in the mucus layer of colon tissue and maintain the immune homeostatic state of small intestine. *Lachnospiraceae*, as the main butyric acid producing bacteria, can promote the increase of butyric acid level in the intestinal tract, and further play a positive role in maintaining intestinal homeostasis by supporting the proliferation of epithelial cells and promoting the epithelial barrier function (34, 73). However, mice may eat their own feces affecting the gut microbiota if multiple mice are kept in the same cage. This is a limitation in our study and the effect of this factor will be considered in future studies.

Spearman’s correlation analysis showed that *Parabacteroides* was significantly positively correlated with inflammatory factors IL-6 and IL-1β, which was consistent with the obvious correlation between *Parabacteroides* with IBDs and many other diseases (70). Anti-inflammatory factor IL-10 were strongly correlated with beneficial bacteria such as *Oscillospira*, *Olsenella*, and *Lactobacillus*. Some *Oscillospira* species could utilize host glycans and secrete important short-chain fatty acid butyric acid. Butyric acid has been proved to prevent inflammation by inducing differentiation of regulatory T cells and downregulating the genes encoding pro-inflammatory cytokines (74). *Lactobacillus* was reported to have a positive effect on suppression the pro-inflammation factors as a probiotic. From the above, we elucidated that taking algal oil may promote the increase of beneficial bacteria to promote the recovery of inflammation.

Optimizing the types and ratio of carbon source and nitrogen source during the cultivation of *Schizochytrium* sp. can affect the DHA production (75). In this study, we used algal oil of *S. limacinum* SR21 fermented with yeast extract and rapeseed meal as nitrogen sources, respectively. The DHA content of the two is similar, but the ratio of saturated fatty acids, especially myristic acid C14:0, was quite different. Myristic acid is a traditional Chinese herbal medicine, there are few studies of the anti-inflammatory effects and regulation of intestinal flora of myristic acid. A study also revealed that myristic acid exerted anti-inflammatory activity by the increasing the production of IL-10 *in vitro* and on TPA-induced ear edema in mice (76). Myristic inhibited the growth of *L. monocytogenes* and influenced cell death when added to dairy products (77). Two algal oils used in this study contain similar DHA/EPA, but differs from C14:0 fatty acid content. RDA analysis also demonstrated the associated between myristic acid and *Bacteroides* and *Enterococcus*. These results suggested that besides n-3 PUFAs, myristic acid might also play an important role in different modulation pattern of gut microbiota for those algal oils.

Diet is one of the most direct and profound influences on gut microbiota, apart from genetics and birth mode (77). The issue of the causal relationship between intestinal flora and disease was complex, it is generally believed that intestinal flora metabolizes the ingested nutrients and produces a wide range of microbial metabolites, which have an important impact on human physiology. In our study, there was no significant difference between the reparation effects on the CS-induced injury mice for these two kinds of oil, but they indeed regulated the bacterial flora in CS-treated mice. We conceived the intestinal flora displayed a faster obvious changes response to the algal oil supplementation. Since it was short-time treatment which not enough to make a significant difference in intestinal tissue physiology. Or it could be explained due to the key fatty acids (DHA or myristic acid) at this dosage was inadequate to show differences. Thus, the relationship between the content of the algal oil, especially, some vital biocompounds, and the regulation of intestinal flora should be investigated in future work. In addition, a limitation of this study is that short branched chain fatty acids (SCFA) were not measured and we will measure it in further studies.

In our study, algal oil regulated the gut microbiome and inflammatory associated biomarker; it might also affect the pathways of metabolites such as short-chain fatty acids and bile acids as modulated microbiota indicated above. Whether the bacteria investigated in this paper are involved in the production of short-chain fatty acids can be further studied. Our research determined that the algal oil from *S. limacinum* SR21 derived from different substrates displayed modulation effects on intestinal flora, which suggested great efforts on optimization of the carbon source or nitrogen source should be made to obtain the superior DHA oil with premium anti-inflammatory and repair the intestinal effect.

## Data availability statement

The data presented in this study are deposited in the NCBI repository, https://www.ncbi.nlm.nih.gov/, accession no. PRJNA882239.

## Ethics statement

This animal experiment was approved by the Animal Ethics Committee of Huazhong Agricultural University (Permission HZAUMO-2021-0170).

## Author contributions

HZ, ZX, XW, and CY designed the experiments. HZ conducted the experiments, analyzed the data, and wrote the manuscript. WC, SC, XW, and CY revised the manuscript. CY and FH acquired the funding and supervised the project. All authors agreed to be accountable for the work.
