# Supplementary material for: Algal oil alleviates antibiotic-induced intestinal inflammation by regulating gut microbiota and repairing intestinal barrier
Source: Front Nutr. 2023 Jan 16;9:1081717. doi: 10.3389/fnut.2022.1081717 (PMC9884693; doi:10.3389/fnut.2022.1081717)

Supplementary Material

# Supplementary tables

**Table.S1 The fatty acid composition and the relative content (%) of two kinds of algal oil.**

| Saturated fatty acid | YE | RSM |
| --- | --- | --- |
| C10:0 | 0.04±0.002 | 0.11±0.002 |
| C12:0 | 0.28±0.003 | 0.08±0.003 |
| C14:0 | 12.8±0.05 | 4.21±0.05 |
| C15:0 | 0.96±0.01 | 0.77±0.01 |
| C16:0 | 29.09±0.04 | 19.42±0.04 |
| C18:0 | 0.39±0.01 | 0.36±0.01 |
| C21:0 | 0.25±0.002 | 0.31±0.002 |
| C22:0 | 0.49±0.03 | 0.74±0.03 |
| C24:0 | 12.66±0.1 | 18.32±0.1 |
| Unsaturated fatty acid | YE | RSM |
| C16:1 | 0.39±0.04 | 0.36±0.04 |
| C18:1n9t | 0.00 | 1.88±0.1 |
| C18:1n9c | 0.18±0.02 | 1.38±0.02 |
| C18:2n6c | 0.00 | 1.35±0.03 |
| C18:3n3 | 0.00 | 0.32±0.03 |
| C20:3n6 | 0.18±0.01 | 0.37±0.01 |
| C20:4n6（ARA） | 0.28±0.01 | 0.55±0.01 |
| C20:5n3（EPA） | 1.28±0.02 | 2.47±0.02 |
| C24:1 | 0.58±0.01 | 0.00 |
| C22:6n6（DHA） | 39.81±0.5 | 46.90±0.5 |

Results are expressed as the mean ± SEM. Fatty acids with a ratio < 0.1 are not shown

**Table.S2** The composition of a standard diet.

| AIN93M | gm% | kcal% |
| --- | --- | --- |
| Fat | 4 | 9.4 |
| Protein | 14.2 | 14.7 |
| Carbohydrate | 73.1 | 75.9 |
| kcal/gm |  | 3.85 |
| Ingredient | gm | kcal |
| Casein, 30 Mesh | 140 | 560 |
| L-Cystine | 1.8 | 7.2 |
| Corn Starch | 495.692 | 1983 |
| Maltodextrin 10 | 125 | 500 |
| Sucrose | 100 | 400 |
| Cellulose | 50 | 0 |
| Soybean Oil | 40 | 360 |
| t-Butylhydroquinone | 0.008 | 0 |
| Mineral Mix S10022M | 35 | 0 |
| Vitamin Mix V10037 | 10 | 40 |
| Choline Bitartrate | 2.5 | 0 |
| Total | 1000 | 3850 |
| *Soluble fiber/carbohydrate | 540.85 |  |
| *Insoluble fiber/carbohydrate | 50.49 |  |

* The total carbohydrate of corn starch was calculated as 85% within 0.1% insoluble fiber, the total carbohydrate of maltodextrin was calculated as 96%. The content of soluble and insoluble fiber in the diet slightly varies on the different batch of the corn starch and maltodextrin.

## Supplementary Figures

**Supplementary Figure S1.**


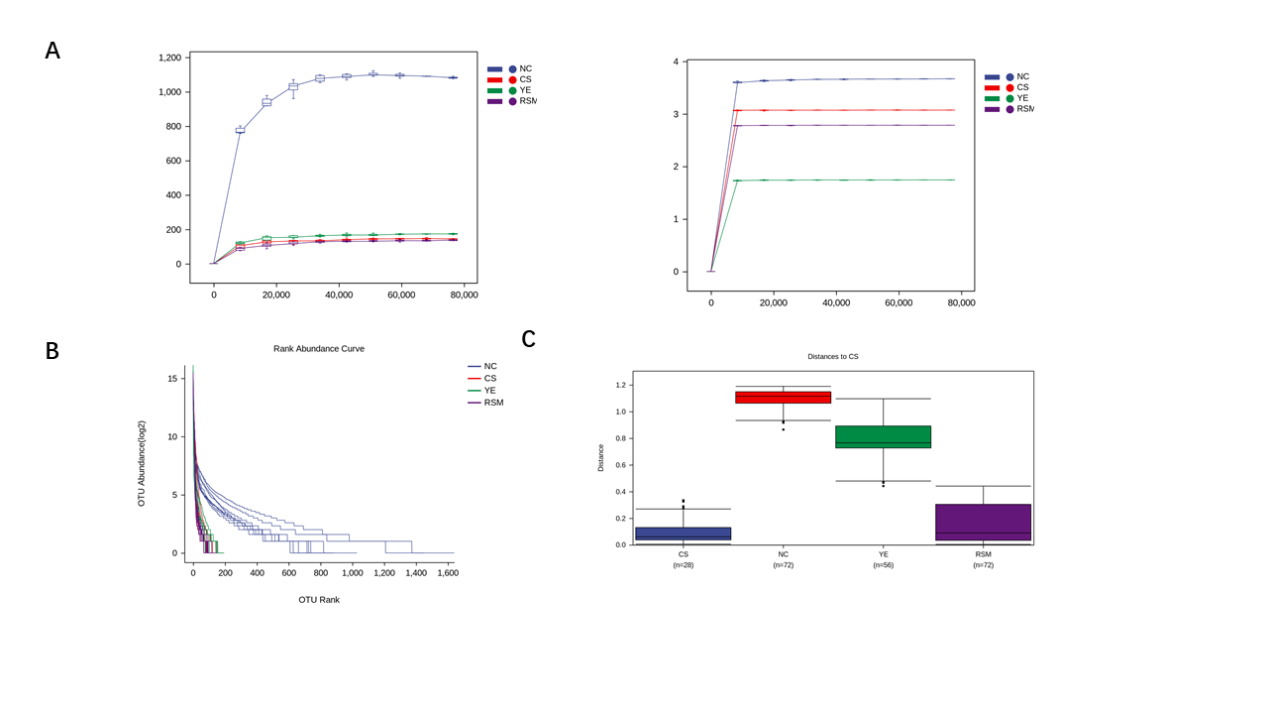


(A) The sparse curve is based on Chao1 and Shannon index types. (B) Abundance grade curve. (C) differences between groups were analyzed based on the weighted Unifrac distance matrix.

**Supplementary Figure.S2**

Metabolic pathway statistics. (Count statistics and relative abundance statistics)


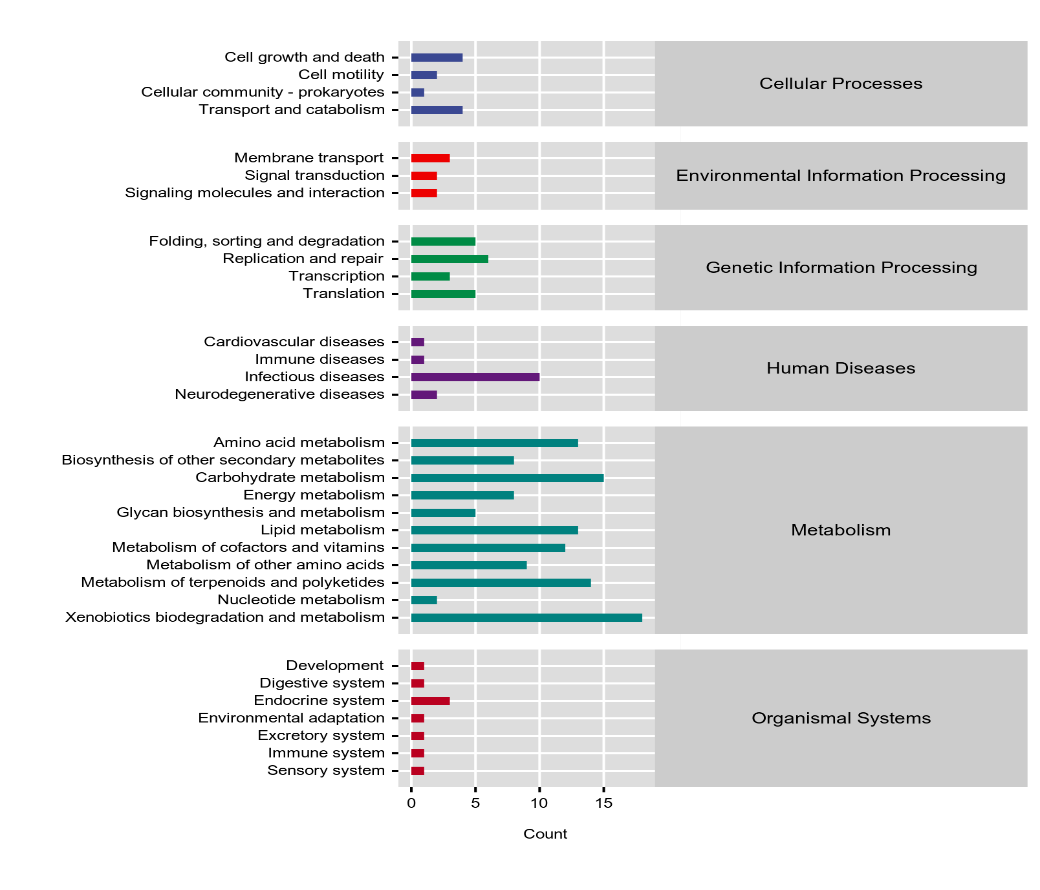


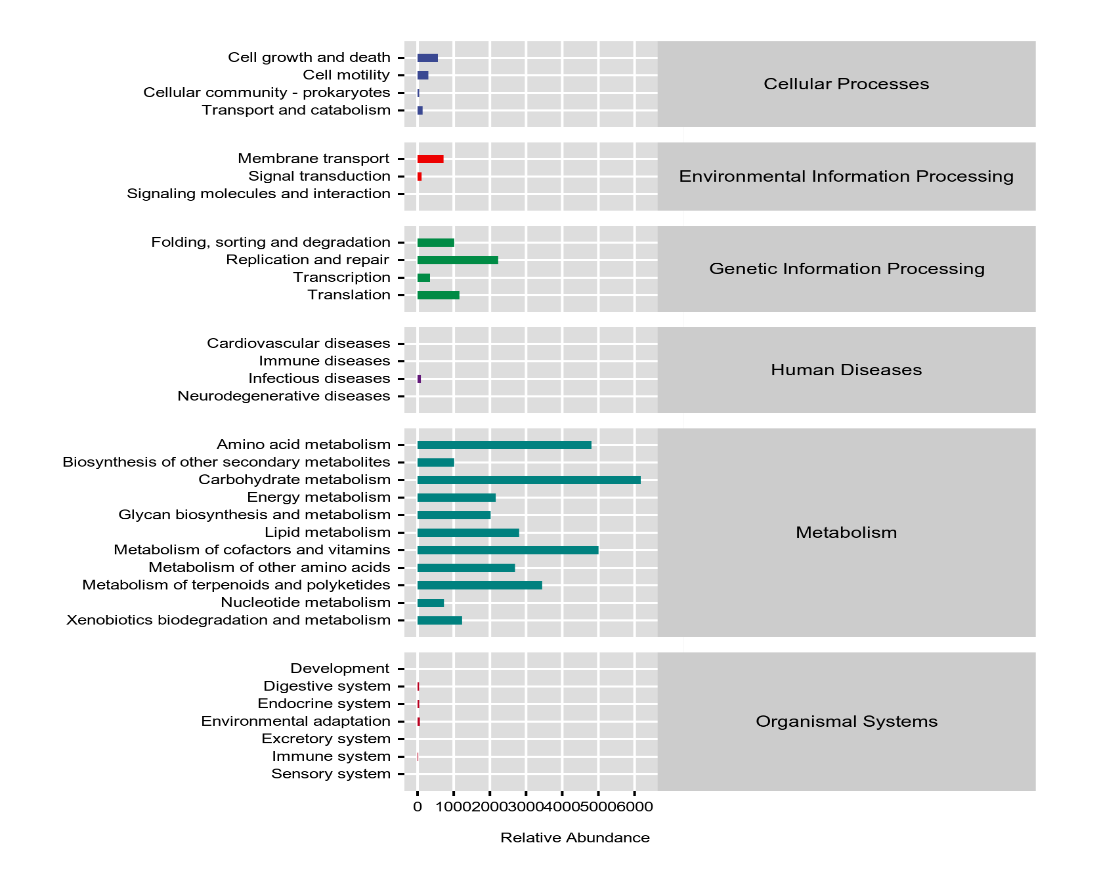

Supplement: Supplementary file 1 [file Table_1.DOCX]
